# Editorial: Genetics and molecular breeding in aquaculture animals

**DOI:** 10.3389/fgene.2022.1071303

**Published:** 2022-11-10

**Authors:** Yue Yu, Alexandre Wagner Silva Hilsdorf, Li Zhou, Qiang Lin, Ze-Xia Gao

**Affiliations:** ^1^ Key Lab of Freshwater Animal Breeding, Ministry of Agriculture and Rural Affairs/Key Lab of Agricultural Animal Genetics, College of Fisheries, Breeding and Reproduction of Ministry of Education/Engineering Research Center of Green Development for Conventional Aquatic Biological Industry in the Yangtze River Economic Belt of Ministry of Education/Engineering Technology Research Center for Fish Breeding and Culture in Hubei Province, Huazhong Agricultural University, Wuhan, China; ^2^ Unit of Biotechnology, University of Mogi das Cruzes, Mogi das Cruzes, Brazil; ^3^ State Key Laboratory of Freshwater Ecology and Biotechnology, Hubei Hongshan Laboratory, The Innovation Academy of Seed Design, Institute of Hydrobiology, Chinese Academy of Sciences, Wuhan, China; ^4^ CAS Key Laboratory of Tropical Marine Bio-Resources and Ecology, South China Sea Institute of Oceanology, Chinese Academy of Sciences, Guangzhou, China

**Keywords:** aquaculture, genetics, genomics, molecular breeding, SNP, genome editing

As a major source of “blue food” (Gephart et al., 2021), aquaculture is an important part of meeting the growing human demand for protein, and is also the fastest growing field of food production in the world (FAO, 2020). Selective breeding offers enormous potential to improve important commercial characters and increase commercial efficiency in aquaculture by providing cumulative and permanent genetic improvement of farmed species. Compared to traditional genetic improvement strategies, molecular marker-assisted selection (MAS) rapidly screens for dominant formations within a population by locating genetic markers that are closely linked to the target trait, which greatly saves time and economic costs to increase breeding efficiency (Collard and Mackill, 2008). During the past few decades, better molecular markers, sequencing techniques and breeding algorithms mean that the mapping of specific genetic loci for economic characters is more efficient and precise. In particular, the advent of high-throughput sequencing technologies has greatly facilitated the rapid development of genomics and molecular biology to analyze the biological and genetic background of quantitative traits, so as to promote the application of aquaculture molecular breeding. The aim of this Research Topic is to collect latest molecular breeding model and/or high-quality research finding on aquaculture genetics and genetic breeding. At present, the Research Topic has collected twelve articles, which are groups in seven different themes (Table 1), and are summarized in detail as follows:

**TABLE 1:** Main research themes included in the Research Topic.

No.	Theme	Species	Main findings	References
1	Genetic diversity	sea urchin *Mesocentrotus nudus*	51,738 biallelic SNPs	Wang et al.
2	Discovery of associated molecular markers and candidate genes for economic traits	oriental river prawn *Macrobrachium nipponense*	NF_k_Bα had a positive regulatory effect on testicular development	Jin et al.
		swimming crab *Portunus trituberculatus*	8 molecular markers for salt-alkali tolerance	Zhang et al.
		Pacific white shrimp *Litopenaeus Vannamei*	489 DEGs	Zhang et al.
3	Linkage maps and QTL analysis	Red swamp crayfish *Procambarus clarkii*	4,878 SNPs and 94 LGs	Guo et al.
		common carp *Cyprinus carpio*	28,416 SNPs and 50 LGs	Zhang et al.
		large yellow croaker *Larimichthys crocea*	9,885 SNPs and 24 LGs	Yu et al.
4	References genome assembly	pedunculate barnacle *Capitulum mitella*	Frist draft genome assembly	Chen et al.
5	Exploratory improvement methods for molecular breeding	Atlantic salmon *Salmo salar*	A cost-saving approach to genotyping	Dagnachew et al.
6	Quantitative genetic basis and genomic architecture of commercial traits	Atlantic salmon *S. salar*	Genetic variation and genetic correlation for smoltification traits	Khaw et al.
		Nile tilapia *Oreochromis niloticus*	Population genetic variation and QTL localization for feed efficiency traits	Barría et al.
7	Functional gene impact on omics	zebrafish *Danio rerio*	22 DEmiRNAs	Domingues et al.

**Theme 1** (Genetic diversity): The estimation of population genetic diversity can not only provide basic information for resource assessment and management, but lay the foundation for germplasm improvement. Wang et al. used GBS (genotyping-by-sequencing) technology to genotype 80 *Mesocentrotus nudus* individuals from five populations along the China’s coast with single nucleotide polymorphisms (SNPs) at the genome-wide level, and systematically described the genetic diversity and population structure of *M. nudus*. The comprehensive results of principal component analysis, admixture and phylogenetic analysis revealed a low genetic differentiation and high genetic connectivity among the five populations, suggesting that timely conservation measures should be taken for wild *M. nudus* resources.

**Theme 2** (Discovery of associated molecular markers and candidate genes for economic traits): Accurate targeting of genetic markers associated with target traits is a crucial prerequisite for molecular breeding. Jin et al. confirmed the inhibitory effect of eyestalk on male development in *Macrobrachium nipponense* by histological observation of gonads. Transcriptome differential analysis of normal prawns (CG), single-side eyestalk ablation prawns (SS), and double-side eyestalk ablation prawns (DS) revealed 1,039, 1,226, and 3,682 differentially expressed genes (DEGs) between CG and SS, SS and DS, and CG and DS, respectively. NF_k_Bα was screened for further functional analysis with qPCR, RNAi and histological observation, as it was the most upregulated gene expressed in DS group. The results indicated that NF_k_Bα had a positive regulatory effect on testicular development in *M. nipponense*, and there was a positive regulatory relationship between NF_k_Bα and insulin-like androgen hormone (IAG), which confirmed the important function of NF_k_Bα in male sexual development of crustaceans for the first time. In the second report, Zhang et al. tentatively explored the salt-alkali tolerance molecular markers in *Portunus trituberculatus* by the bulked segregant analysis (BSA) strategy. Extreme populations of salt-alkali tolerance or intolerance were distinguished to construct two DNA mixing pool libraries. After comparison and association analysis, eight molecular markers that showed significantly related to phenotype were selected, including five SNPs and three indels. For another global crustacean species, *Litopenaeus Vannamei*, Zhang et al. conducted a comparative transcriptome approach to uncover the genetic mechanisms of resistance to *Vibrio parhaemolyticus* (VP_AHPND_), one of the major pathogens of acute hepatopancreatic necrosis disease in *L. vannamei*. After the VP_AHPND_ challenge test, three resistant families and three susceptible families were picked out from 95 families for transcriptome sequencing to construct gene expression profiles. Differential gene expression analysis screened 489 DEGs, of which 19 genes were successfully verified in the offspring lines, underlining the accuracy of these markers.

**Theme 3** (Linkage maps and QTL analysis): Genetic linkage mapping and QTL localization are important tools for marker-assisted selection for breeding because of their ability to identify molecular markers or candidate genes associated with economic traits (Yu et al., 2015; Peng et al., 2016). Three reports in this theme attempted to develop genetic linkage maps for three economically important species. Guo et al. used 2b-RAD sequencing technology to construct a high-density genetic linkage map for a red swamp crayfish (*Procambarus clarkii*) with full-sib family containing 4,878 SNP markers and 94 linkage groups (LGs). Using this genetic map, researchers located the growth- and sex-based QTLs of *P. clarkii* for the first time. Ultimately, 28 QTLs associated with multiple growth traits were identified in nine LGs and a sex-determining major locus was identified in LG20. Notably, the majority of the identified SNPs were female heterozygotes, suggesting that *P. clarkii* may have a ZZ/ZW sex determination system. Similarly, Zhang et al. used a 250 K SNP array to genotype the full-sib F1 family of mirror carp (*Cyprinus carpio*), resulting in the construction of a high-density and high-resolution genetic linkage map for common carp containing 28,416 SNP markers with 50 linkage groups. In this study, a total of 17 QTLs related to feed conversion efficiency (FCE) were mapped on four LGs, and nine candidate genes related to carp FCE involved in multiple biological processes were further identified. In another research, Yu et al. constructed consensus and sex-specific (female and male) genetic linkage maps for large yellow croaker (*Larimichthys crocea*) using samples from an F1 family produced by crossing two distant strains (Daiqu female × Mindong male), which contained 20,147, 11,838, and 11,684 SNPs respectively. Then, the genetic linkage map with 9,885 SNPs and 24 LGs was adapted and integrated based on the physical map of large yellow croaker, and the recombination patterns of each linkage group and the recombination rate of the sex-specific integration map were analyzed in detail. Since previous SNP linkage maps were constructed only for the Mindong strain, this hybrid strain-based linkage map effectively amplified the genetic molecular markers in large yellow croaker.

**Theme 4** (Reference genome assembly): Fully assembled and annotated genome will contribute to the study of taxonomic identification, phylogenetic analysis, physiological mechanism and species protection, *etc.* Chen et al. used PacBio and Hi-C sequencing data to construct a first draft of the genome of the pedunculate barnacle (*Capitulum mitella*) with a size of 463.09 Mb, and generated 16 chromesomes anchored by the contigs. After exhaustive annotation of the genome, the authors also carried out an evolutionary and phylogenetic analysis between *C. mitella* and nine other species based on amino acid sequences.

**Theme 5** (Exploratory improvement methods for molecular breeding): Innovative improvement of genotyping methods may bring great improvement to aquaculture molecular breeding. Dagnachew et al. expected to reduce the breeding costs of aquaculture genomic selection by grouping individual samples from reference populations into DNA sample poolings for genotyping, rather than for each individual. Using databases from two salmonid alphavirus-challenged Atlantic salmon (*Salmo salar*) populations, the researchers assessed the impact of the number of DNA poolings in the reference population vs. sequencing coverage on the accuracy of calculating allele frequencies, SNP effects and genomic breeding values (GEBV). A reasonable DNA pooling strategy (i.e., the number of individuals merged in the pool) can provide computational accuracy with little or no loss compared to individual genotyping. And the need for sequencing depth will decrease as the number of DNA pools increases.

**Theme 6** (Quantitative genetic basis and genomic architecture of commercial traits): High mortality rates are limiting the Atlantic salmon industry globally. Smoltification is a series of behavioural, developmental and physiological changes during the transfer of Atlantic salmon from freshwater to seawater, which determine the ability of Atlantic salmon to adapt to the seawater environment and therefore urgently needs to be exhaustively investigated. Khaw et al. explored genetic variation of different several phenotypic traits associated with smoltification status by controlling the light regime to simulate seasonal sunshine, and assessed the genetic correlation of smoltification traits between two different age groups from the same family. Conclusion of the study emphasises the importance of the correct choice of time point for measuring phenotypes and fish transfer to seawater, as the smoltification period has different temporal progression between age groups. In the next report, Barría et al. make an outstanding contribution to genetic breeding for feed efficiency in Nile tilapia (*Oreochromis niloticus*). The genetic structure of the population feed efficiency traits was analyzed in detail, suggesting that feed efficiency traits have significant heritability to be used for genetic improvement in Nile tilapia. In addition, genome-wide association analysis (GWAS) was used to locate QTLs for four feed efficiency traits.

**Theme 7** (Functional gene impact on omics): Domingues et al. combined computer-assisted sperm analysis and transcriptome analysis to demonstrate that overexpression of growth hormone (*gh*) genes altered the microRNA expression profile of zebrafish testis, thus reducing the sperm motility and reproductive potential. The researchers compared the microRNA transcriptomes of *gh*-transgenic zebrafish (F0104 strain) and non-transgenic zebrafish. 22 differentially expressed microRNAs were monitored, and their functions were adequately interpreted or predicted. Notably, these identified candidate microRNAs may be able to serve as cross-species biomarkers for the validation of male fertility. This study provides the first microRNAome perspective to explain that miRNAs act as direct players in mediating the adverse effects of GH on male reproductive function.

In conclusion, molecular marker-assisted breeding, particularly genomic selection breeding, has great potential to accelerate the rate of genetic gain for excellent characters, which is widely recognized by aquaculture breeders. This Research Topic provides an excellent Research Topic of comprehensive content on aquatic genetics and molecular breeding. We expect that the continuous improvement of new and high-tech in the future will further accelerate the process of molecular breeding of economical aquatic species.
